# Transcriptome-derived variants in milk reveal host response signatures to subclinical intramammary infection in Holstein cattle

**DOI:** 10.1186/s40104-026-01411-0

**Published:** 2026-05-17

**Authors:** Alice Vanzin, Vittoria Bisutti, Ángela Cánovas, Alessio Cecchinato, Luigi Gallo, Diana Giannuzzi, Sara Pegolo

**Affiliations:** 1 Department of Agronomy, Food, Natural Resources, Animals, and Environment (DAFNAE), University of Padova, Viale Dell’Università 16, Legnaro, PD 35020 Italy; 2 Centre for Genetic Improvement of Livestock, Department of Animal Biosciences, University of Guelph, 50 Stone Road East, Guelph, ON N1G 2W1 Canada

**Keywords:** Dairy cows, Intramammary infection, Transcript variants, Transcriptome

## Abstract

**Background:**

Beyond conventional transcriptome profiling, RNA-sequencing (RNA-Seq) enables the discovery of variants within expressed genes linked to complex traits such as mastitis resistance or susceptibility. In this study, RNA-Seq was performed on milk somatic cells from uninfected Holstein cows with no history of mastitis (Neg, *n* = 9) or with subclinical intramammary infections (sIMI) caused by *Prototheca* spp. (P+ ,* n* = 11) or *Streptococcus agalactiae* (Sa+ , *n* = 11). The objective was to identify transcriptome-derived sequence variants detectable under specific microbiological conditions that may contribute to the modulation of host transcriptional responses. By integrating these transcript-derived variants with quantitative trait locus (QTL) annotations and enrichment analyses, we aimed to highlight genomic regions functionally associated with mastitis susceptibility or resilience.

**Results:**

Using the CLC Genomic Workbench, a total of 306,440, 264,132, and 246,777 unique variants were detected in Neg, P+ , and Sa+ groups, respectively, with SNPs being more abundant than INDELs. High-impact variants were identified in immune-related genes (*TNIP1*, *TNIP3*, *IL10RB*, *IL2RA*, *IL15RA*), suggesting post-transcriptional modulation of immune and inflammatory responses under different microbiological conditions. Variants in non-coding regulatory regions indicate that transcriptional control may also contribute to host susceptibility. QTL enrichment using GALLO showed consistent associations with milk production traits, while clinical mastitis QTLs were specifically enriched in P+ , reflecting context-dependent regulation of immune responses.

**Conclusions:**

These findings provide new insights into the molecular basis of host responses to intramammary infections and support the value of a transcriptome-driven approach for variant discovery. Variants identified in immune-related genes and regulatory regions suggest that both immune gene modulation and post-transcriptional regulation may contribute to susceptibility to sIMI. Upon further functional validation, these findings may inform precision breeding strategies to enhance mastitis resilience in dairy cattle.

**Supplementary Information:**

The online version contains supplementary material available at 10.1186/s40104-026-01411-0.

## Background

Developing effective mastitis control strategies is a key priority for bovine dairy farming. This disease, primarily caused by infectious agents [1], remains a major challenge for the sector, with a negative impact on both productivity and animal welfare [2]. In addition, non-bacterial causes of intramammary infection (IMI) are also gaining increased attention; for example, algae of the genus *Prototheca* are naturally resistant to conventional treatments, thereby making protothecal mastitis particularly challenging to manage [2–4].

Beyond clinically confirmed IMI, subclinical IMI (sIMI) also poses an insidious challenge, as the absence of clear manifestation significantly delays diagnosis and allows the infection to spread throughout the herd [5]. To date, IMI represents the primary reason for antibiotic use in adult dairy cows [2]. In light of the growing concern about antibiotic resistance [6], both in livestock and human health, enhancing the naturally occurring resistance of cattle to invading pathogens may represent a promising strategy to reduce the reliance on such therapeutic treatments.

In this scenario, preventive strategies, selective breeding, and good environmental practices could represent a valuable opportunity to limit the impact of this disease. Exploitable genetic variability for IMI exists [7], even for pathogen-specific resistance [8], providing an opportunity for genetic improvement. However, the overall resistance to mastitis is characterized by a low heritability (≤ 0.05) [8, 9], which limits the success of traditional pedigree-based genetic selection strategies [10]. In such contexts, genomic approaches that focus on increasing the frequency of favorable alleles across key genomic regions represent a promising alternative [10, 11]. These strategies are particularly well-suited for complex, polygenic traits like mastitis resistance, where genetic architecture involves multiple loci with small additive effects [12]. The constant improvement of high-throughput sequencing platforms, combined with their increasing cost-effectiveness, has enabled the identification of numerous sequence variants associated with IMI resistance, which have been widely used as molecular markers in genomic predictions and genome-wide association studies [10, 11, 13–17]. To this end, RNA-sequencing (RNA-Seq) represents an efficient strategy to detect coding sequence variants such as single nucleotide polymorphisms (SNPs) and short sequences insertions and deletions (INDELs) in the expressed regions of various tissues, representing a cost-effective alternative to the whole genome sequencing, reducing the complexity of data, and increasing the chance to find functional causative variants [18–24]. Importantly, this approach enables the identification of sequence variants detectable in expressed transcripts under specific conditions that may influence transcriptional regulatory responses, thereby providing greater biological relevance. RNA-based sequence variants discovery has successfully been applied to livestock, to study important production traits such as feed efficiency, health, fertility, and meat quality traits [23, 25–32]. However, to the best of our knowledge, no studies focusing on sIMI have applied this technique, relying instead primarily on SNP-chip or DNA-based approaches.

In light of this, in this study we aimed to identify transcriptome-derived sequence variants detectable in milk somatic cell RNA of Holstein cattle across different microbiological conditions of the mammary gland. In particular, we focused on *i*) animals with a natural sIMI due to *Prototheca* spp. (P+), *ii*) animals with a natural sIMI due to *Streptococcus agalactiae *(*S. agalactiae,* Sa+), and *iii*) uninfected controls with negative bacteriological results (Neg).

By integrating these transcript-derived variants with QTL mapping, we aimed to identify functionally relevant genomic regions associated with mastitis susceptibility or resilience. This dual-layer approach provides novel insights into transcriptional variation associated with different microbiological conditions and may help prioritize candidate loci for further functional and genomic validation.

## Methods

### Experimental design and sample collection

Based on a survey conducted by the Istituto Zooprofilattico Sperimentale delle Venezie (IZSVe, Legnaro, PD, Italy) to assess the prevalence of the most common pathogens responsible for mastitis in the Veneto region, a commercial herd of 450 lactating Holstein cows located in Verona province (Veneto region, Italy) with prevalence of *S. agalactiae* and *Prototheca* spp. was selected to conduct the study. All cows of the herd were housed in free stall and fed total mixed ration primarily composed of corn silage, sorghum silage, and concentrates. Drinking water was provided through automatic water bowls, and milking was performed twice daily. Further details on management and farming conditions can be found in Bisutti et al. [33].

To be enrolled in the study, animals had to meet specific criteria: *i*) absence of clinical signs of infection or inflammation, *ii*) no previous documented history of mastitis, and *iii*) no ongoing antibiotic treatments or anti-inflammatory medications. Additionally, to minimize potential confounding effects related to postpartum metabolic diseases, only multiparous cows having > 120 d in milk were considered. According to these criteria, at time zero (T0) an initial group of 188 animals was selected and a microbiological screening was carried out on the composite milk collected by sterile manual milking. Results allowed the selection of three groups of animals: negative to microbiological test (Neg, *n* = 9), positive to *Prototheca* spp. (P+, *n* = 11), positive to *S. agalactiae* (Sa+, *n* = 11). Animals with a co-infection were excluded from the study. After 15 d (Time 1, T1), animals belonging to these groups were sampled again. Regarding the present study, at T1 three aliquots of individual composite milk each were collected by sterile manual milking for *i*) confirmation of the microbiological status; *ii*) assessment of milk quality and udder health traits; *iii*) RNA-Seq analysis of milk somatic cells.

For both the time points, individual composite milk destined to microbiological analysis and RNA-Seq was collected in 50-mL sterile tubes, in accordance with the National Mastitis Council guidelines [34]. The teats were cleaned with commercial disinfectants, dried, and finally washed with 70% ethanol; the individual composite milk was collected by sterile manual milking, after discarding the first streams of foremilk from each quarter. Milk samples destined to assessment of milk composition and udder health traits were collected in provided tubes supplemented with the antimicrobial Bronopol 2-bromo-2-nitro-1,3-propanediol (Merck, Darmstadt, Germany), following the same procedure. Then, all samples were stored at 4 °C and transported, maintaining the cold chain, to the different laboratories for the analyses.

### Microbiological analysis, milk composition and udder health traits

Microbiological analyses were conducted at the IZSVe. After the sampling, milk aliquots were transported to the laboratory, where they were frozen (−20 °C) and processed within the following 3 d. For the analyses, 10 µL of milk was plated onto three different selective media: Baird-Parker agar with rabbit plasma fibrinogen (BP-RPF; Biokar Diagnostics, Beauvais, France), thallium crystal violet toxin agar (TKT; IZSVe internal production), and *Prototheca* isolation medium (PIM; IZSVe internal production). Suspected *S. agalactiae* colonies were confirmed using the Christie–Atkins–Munch-Petersen test [34] after 24 h of incubation. In parallel, *Prototheca* isolation medium plates were examined at 24, 48, and 72 h, and suspected colonies were confirmed using the wet mount method [34]. More details are described in Pegolo et al. [35].

Analyses of milk composition, somatic cell count (SCC) and differential somatic cell count (DSCC) were run at the Milk Quality Laboratory of the Association of Breeders of Veneto (ARAV, Padova, Italy) within 24 h of sampling. Milk composition, given by fat, protein, casein, lactose, and urea, was determined with the FT6000 Milkoscan infrared analyzer (Foss Electric A/S); SCC and DSCC were measured with the Fossomatic™ 7 DC analyzer (Foss A/S, Hillerød, Denmark). The SCC was log-transformed in somatic cell score (SCS) to achieve normality [36].

### RNA extraction, library preparation and sequencing of milk somatic cells

The milk aliquots collected at T1 and destined for RNA-Seq analysis were transported to the milk laboratory of the Department of Agronomy, Food, Natural Resources, Animals and Environment (DAFNAE, University of Padova, Legnaro, PD, Italy). Milk somatic cell fraction was used to perform RNA-Seq analysis as described by Cánovas et al. [37]. In detail, RNA from milk somatic cells was extracted and purified using a NucleoSpin miRNA kit (Macherey Nagel, Düren, Germany), following the combined protocol with TRIzol lysis with small and large RNA in one fraction (total RNA). Then, the concentration and quality of the extracted RNA were assessed using the Agilent 2100 Bioanalyzer (Santa Clara, CA, USA). The RNA samples were subsequently shipped on dry ice to the Nuova Genetica Italiana (NGI, Como, Italy) facility for library preparation and sequencing. The RNA-Seq libraries were prepared starting from 500 ng of total RNA using the MGIEasy RNA Library Prep Set V3.1 (MGI Tech Co., Ltd., Shenzen, China), according to the manufacturer’s protocol. Finally, RNA-Seq was performed on a DNBSEQ-G400 high throughput machine (MGI Tech Co., Ltd.) with a paired-end approach using the DNBSEQ-G400 sequencing kit (MGI Tech Co., Ltd., Shenzen, China). Additional information about RNA extraction, library preparation, and RNA-Seq are reported in Bisutti et al. [33].

### RNA‑Seq data processing and variants calling

The RNA-Seq data were used to identify SNPs and INDELs variants within the transcriptome using the CLC Genomics Workbench 23.0.5 (QIAGEN, Aarhus, Denmark), as previously described [23, 29, 38]. Firstly, raw sequences were trimmed according to the following parameters: quality limit = 0.05, maximum number of ambiguities = 2, maximum length = 150 bp. Quality control was then carried out to evaluate Guanine-Cytosine (GC) content, Phred score, and the presence of overrepresented sequences [37]. Then, as previously described in Lam et al. [24], the trimmed sequences from all samples within each experimental group were aligned to the bovine reference genome (*Bos taurus* ARS-UCD1.3) to generate a unique alignment for each condition (Neg, P+, and Sa+). This strategy increased overall coverage, including in regions with low expression, while reducing the impact of random technical noise on variant detection. The three different alignments were individually used as input for the variant calling step using the *Fixed Ploidy Variant Detection* tool. To reduce false positives arising from sequencing errors or sporadic low-frequency events the following parameters were set: required variant probability = 90%; ignore broken pairs; minimum coverage = 10; minimum count = 2; minimum frequency = 10%. The base quality filter was performed using a minimum quality for the central base and for the five upstream and downstream bases of 20 and 15, respectively as described by Cánovas et al. [23]. After this step, three tracks (Neg, P+, and Sa+ track) reporting the variants detected in each experimental group were obtained. Subsequently, all the variants included in the three tracks were merged into one using the *Identify Shared Variants* tool setting the *frequency threshold* parameter to 0%; variants that were common to two or more conditions and variants identified as the reference allele were then excluded, as variants uniquely identified in one of the groups were the interest of the study. The final track containing only the variants unique of a single condition was used to predict the functional consequences: *Amino Acid Changes* and *Predict Splice Site Eff*ect tools were used to detect variants resulting in amino acid change and/or variants falling within potential splice sites, respectively. Importantly, because transcriptome-derived variants can only be detected in expressed transcripts, those identified here reflect changes observable under specific microbiological conditions. Accordingly, the absence of a variant in a given group does not necessarily indicate its true genomic absence.

Additionally, functional effects of the identified variants were assessed using the *Variant Effect Predictor* (VEP, https://www.ensembl.org/Tools/VEP) to obtain information about the genomic location of the variants (i.e., upstream of a transcript, in coding sequence), consequence and impact on the protein sequence (high, moderate, modifier, low), and SIFT score [39, 40]. Variant impact ‘*High*’ means variant resulting in high or disruptive impact in the protein that would lead to protein truncation, loss of function, or tissue nonsense, mediated delay; ‘*Moderate*’ means a non-disruptive variant that may change protein effectiveness; ‘*Modifier*’ means variant affecting non-coding genes, where predictions are difficult or the impact is unknown; ‘*Low*’ means variant that is assumed to be mostly harmless or unlikely to change protein behaviour [40]. Finally, the identified variants were integrated with differential expression results obtained from the same RNA-Seq dataset previously described for genes [33] and transcripts [41]. This integration allowed the identification of variants located within differentially expressed genes (DEGs) and differentially expressed transcripts (DETs), thereby prioriotizing variants occurring in genomic regions with transcriptional changes associated with the different microbiological conditions.

### QTL annotation and enrichment analysis

The QTL classes and traits annotation and enrichment were performed using the R package Genomic functional Annotation in Livestock for positional candidate LOci (GALLO) [42]. The genomic coordinates of all the SNPs and INDELs resulting with *high*, *moderate*, or *modifier* predicted impact were used as input, together with the QTL.gff annotation file downloaded from the cattle QTL Database (https://www.animalgenome.org/cgi-bin/QTLdb/index). The QTL annotation was performed using an interval of 1,000 bp up and down-stream of each variant coordinate. Chromosome-based enrichment analysis was performed using the *qtl_enrich* function to assess the significantly overrepresented QTLs annotated around the candidate variants. The false discovery rate (FDR) correction for multiple testing was applied. A QTL trait was considered enriched with an FDR < 0.05.

## Results

### Animals

The animals enrolled in the study accounted for a mean of 235 d in milk and a mean parity of 2.5. On average, milk yield was 26.97 (± 9.011) kg/d and its composition was 2.19% ± 0.741% fat, 3.493% ± 0.291% protein, 2.727% ± 0.265% casein, 22.339 (± 4.539) mg/100 mL urea, and 4.506% ± 0.444% lactose. Milk had an average pH of 6.455 (± 0.082), SCS of 5.604 (± 2.559), DSCC of 64.639% ± 13.421%. Descriptive statistics are reported in Table S1. More details about composition are reported in Bisutti et al. [33].

### Variant calling from RNA-Seq data

**Fig. 1:**
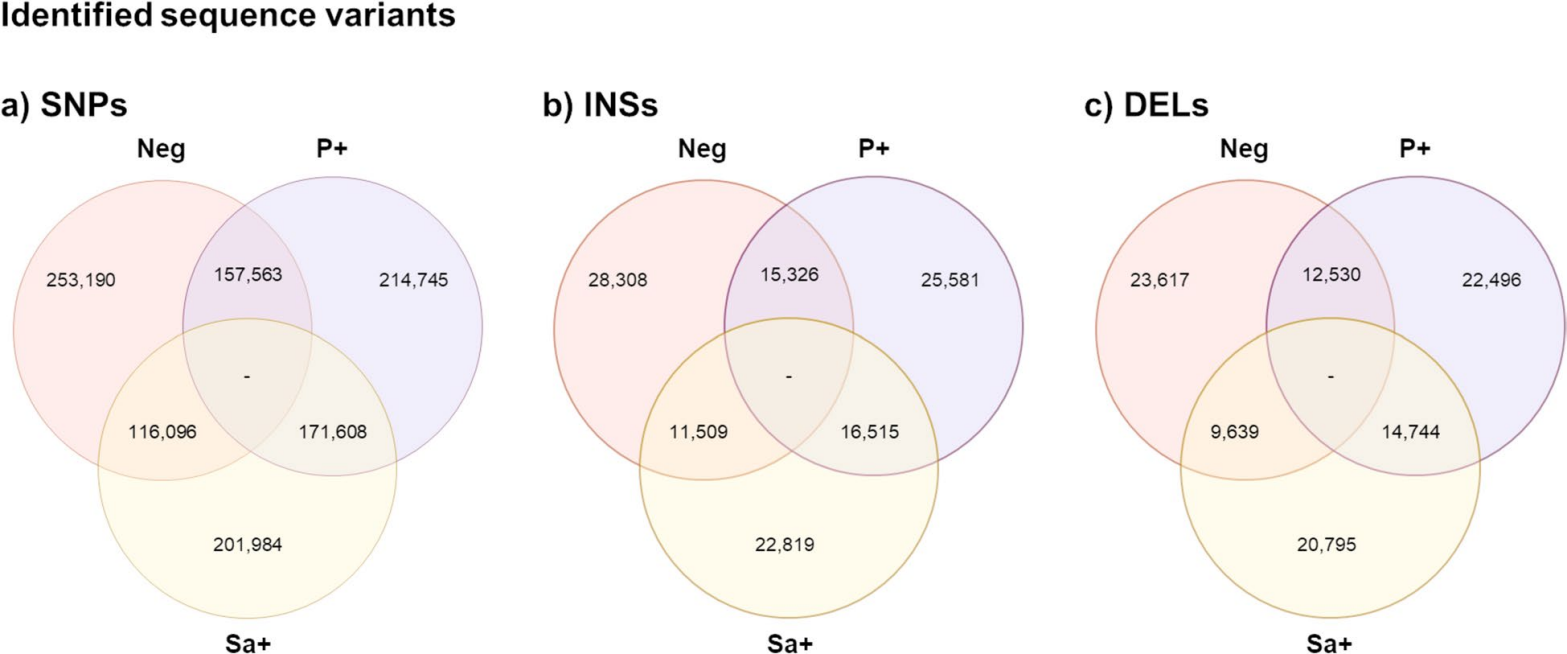
Identified sequence variants. Total single nucleotide polymorphisms, SNPs (**a**), insertions, INSs (**b**); and deletions, DELs (**c**) identified as unique or shared across the different microbiological conditions. Neg, Negative; P+, positive to *Prototheca* spp.; Sa+, positive to *Streptococcus agalactiae*. The variants common to all the conditions were not considered

The RNA-Seq data of 31 samples showed an average of 119 M ± 49.8 M paired-ends reads/samples of 150 bp, of which the 92% mapped to the ARS.UCD1.3 bovine reference genome. After the mapping and the alignment, the variants detection step allowed to identify 1,345,001 variants, of which 1,115,186 were SNPs, 120,058 were insertions, and 103,821 were deletions. The distribution of the variants across the microbiological conditions is shown in Fig. 1. The variants common to all the conditions were not considered.

### Condition-specific detected variants

#### Negative group

A total of 305,115 variants were uniquely detected in negative samples, where 82.99% were SNPs, 9.28% were insertions and 7.73% were deletions; among them, 122 SNPs, 97 insertions, and 54 deletions were predicted to affect splicing, as they fell within putative splice sites (Table S2).

The VEP analyses revealed 251,090 SNPs, 28,194 insertions, and 23,539 deletions having at least one predicted effect with high, moderate, or modifier impact. These variants overlapped 21,628 annotated genic regions, comprising 16,128 protein-coding genes, 3,952 long non-coding RNA (lncRNA), and 484 micro-RNA (miRNA). Several high-impact variants co-localized with genes involved in immune function (e.g., *IL15RA*,* IL2RA*,* IRF6*,* MYPC1*). All high-impact variants uniquely identified in the P+ group, along with their predicted consequences and associated genes, are listed in Table S3.

The majority of both SNPs (73%) and INDELs (75% and 74%) were predicted to be intronic variants with a modifier effect. Among the variants located in coding regions, 41% of the SNPs were classified as missense variants, while 75% of the INDELs were predicted to cause changes in the translational reading frame.

#### Prototheca spp.-positive group

The analysis of P+ group identified a total of 262,822 uniquely detected variants. Among these, 81.75% were SNPs, 9.73% were insertions, and 8.56% were deletions. A subset of these variants (90 SNPs, 99 insertions, and 63 deletions) were predicted to impact splicing, as they were located within putative splice sites (Table S4).

The VEP analyses predicted 213,295 SNPs, 25,472 insertions, and 22,423 deletions with at least one predicted effect with high, moderate, or modifier impact. The identified variants overlapped 19,727 annotated genic regions, of which 14,654 were protein-coding genes, 3,500 were lncRNAs, and 400 were miRNAs. As in the negative group, high-impact variants were found to co-localize with immune-related genes such as *IKZF3*, *CX3CL1*, *BOLA*-*DQB*, *TNIP1*, *IL10RB*, *TLR6.* The complete list of high-impact variants uniquely detected in the P+ group, including their predicted consequences and affected genes, is provided in Table S5.

Most of the SNPs (75.2%) and INDELs (75.3% and 75.4% insertions and deletions, respectively) were classified as intronic variants with a predicted modifier effect. Among the variants located in coding regions, 43% of the SNPs were classified as missense variants. Approximately 66% of the insertions were classified as frameshift variants, 2% as protein-altering variants, and 4% were predicted to generate premature stop codons. Deletions were predominantly classified as frameshift variants (84%).

#### S. agalactiae-positive group

A total of 245,598 variants were uniquely detected in Sa+ samples. These included 82.27% of SNPs, 9.29% of insertions, and 8.46% of deletions. Among them, 94 SNPs, 45 insertions, and 46 deletions were predicted to impact splicing, being located within putative splice sites (Table S6).

The VEP analyses revealed 200,415 SNPs, 22,721 insertions, and 20,708 deletions, each with at least one predicted effect categorized as high, moderate, or modifier impact. A total of 20,111 genes co-localized with the identified variants, including 15,285 protein-coding genes, 3,458 lncRNAs, and 432 miRNAs. Consistent with the other groups, several variants predicted to have high impact co-localized with genes associated with immune pathways (i.e., *IRF6*,* PDE4D*,* TNIP3*), and they are all reported in Table S7.

In line with the the variants specific to Neg and P+, a large proportion of SNPs (74.3%) and INDELs (75.5% and 75.2% for insertions and deletions, respectively) were predicted to be intronic variants with a modifier effect. When focusing on coding-region variants, 44% of the SNPs were classified as missense variants. Among insertions, 75% were classified as frameshift variants, 2% were predicted to generate novel stop codons, and 1% were expected to cause the loss of start codons. Regarding deletions, up to 73% were classified as frameshift variants, and 1% were predicted to result in the loss of start codons.

### QTL annotation and enrichment analysis

The QTL annotation identified 68,953, 63,014, and 57,078 QTLs within a 2 kb interval (1 kb up- and down-stream) of the candidate markers for Negative, P+, and Sa+ group, respectively.

**Fig. 2:**
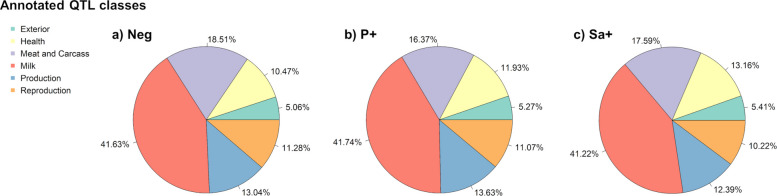
Annotated QTL classes. Percentage (%) of annotated QTL classes co-localized with variants unique to negative (Neg) (**a**), *Prototheca* spp.-positive (P+) (**b**), and *Streptococcus agalactiae*-positive (Sa+) groups (**c**)

Most of the condition-specific detected variants were predominantly co-localized with the *Milk* QTL class, accounting for 41.63% of QTLs in Neg, 41.74% in P+, and 41.22% in Sa+. Within this class, most QTLs overlapping the candidate regions were associated with milk fat yield and milk fat percentage across all groups. The *Meat and Carcass* QTL class ranked second in terms of variants co-localization, with 18.51% in Neg, 16.37% in P+, and 17.59% in Sa+. The third most frequently annotated QTL class was *Production* for both the Neg (13.04%) and P+ (13.63%) groups, whereas for the Sa+ group it was *Health* (13.16%). Notably, the *Health* class ranked fourth in the P+ group (11.93%) and fifth in the Neg group (10.47%). Within the *Health* QTL class, somatic cell score was the second most frequently annotated trait in all groups. Figure 2 presents the percentages of each annotated QTL class across the different groups. All QTL traits annotated within the candidate intervals for each QTL class are reported in Tables S8–S10.

To overcome the bias due to the overrepresentation of some traits in the cattle QTL database, the QTL enrichment analysis was performed. A total of 184 distinct traits resulted enriched (FDR < 0.05) across the groups: 76 in Neg, 80 in P+, and 108 in Sa+ (Tables S11–S13). The positive groups (P+ and Sa+) accounted for 108 exclusively enriched traits; among these, 19 were shared, while 35 and 54 were uniquely enriched in the P+ and Sa+ groups, respectively. The traits exclusively enriched in the Neg group were 36. Traits involving QTLs located on different *Bos taurus* chromosomes were considered distinct.

#### Negative group enriched QTLs

**Fig. 3:**
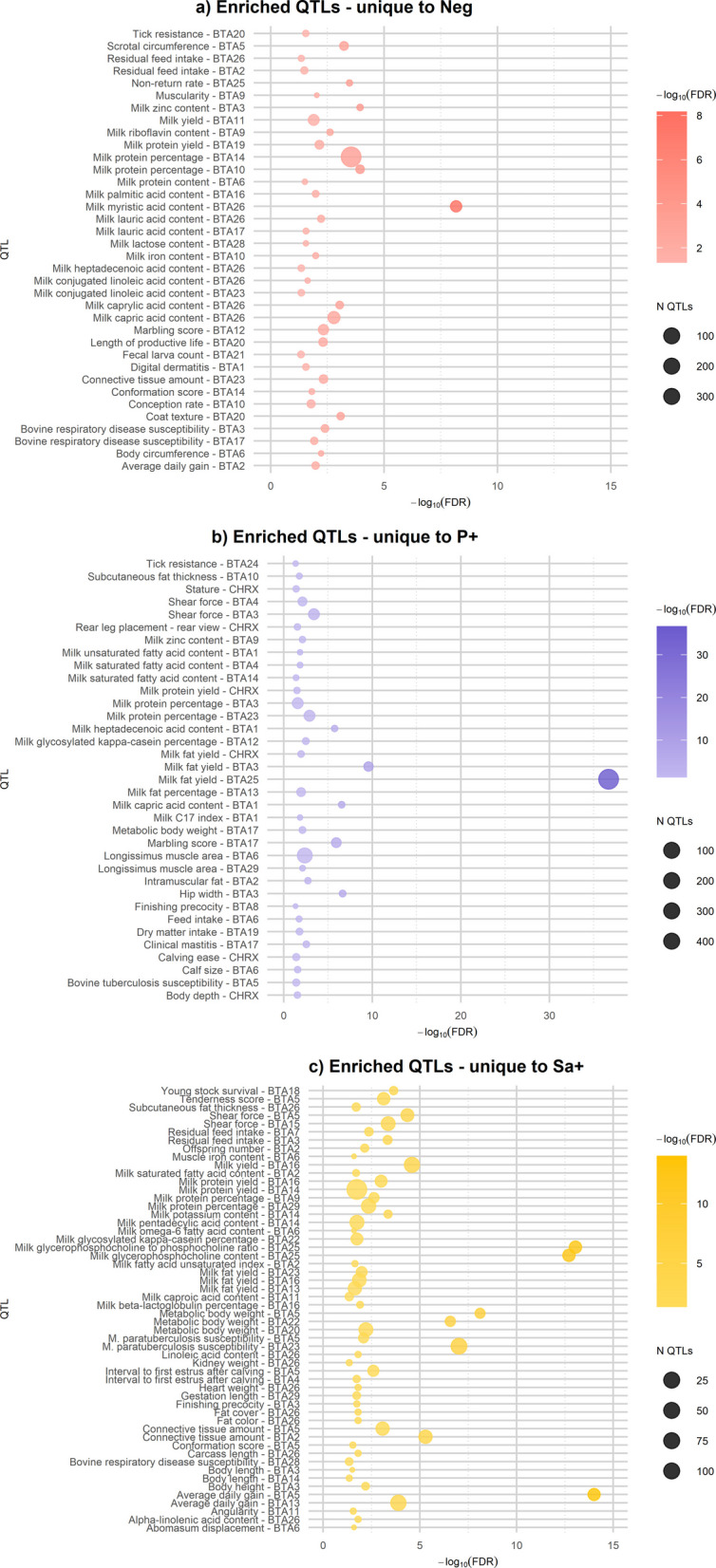
Enriched QTLs. Significantly enriched (FDR < 0.05) QTL traits co-localized with variants unique to negative (Neg), *Prototheca* spp.-positive (P+), and *Streptococcus agalactiae*-positive (Sa+) groups. The area of the bubbles represents the number of observed QTLs for that trait. The *x*-axis shows the −log(*P*_adj_-value) scale, where *P*_adj_ refers to FDR. **a** QTL traits and the corresponding *Bos taurus* chromosomes (BTA) exclusively enriched in Negative group. **b** QTL traits and the corresponding BTA exclusively enriched in *Prototheca* spp.-positive group. **c** QTL traits and the corresponding BTA exclusively enriched in *Streptococcus agalactiae*-positive group

The QTL traits exclusively enriched in the Neg group were 36, as reported in Fig. 3a. Most of these traits (18) belonged to the *Milk* QTL class, particularly related to milk fat and fatty acid content, including milk fat percentage, milk fat yield, milk auric acid content, and milk palmitic acid content. *Milk* QTL class-related traits such as protein, lauric acid, lactose, and iron content were uniquely enriched in the negative group and were not found in any other group, even on different chromosomes. Within the *Health* class, four distinct traits were enriched (FDR < 0.05), including bovine respiratory disease susceptibility (in *Bos taurus* autosome, BTA, 3 and 17), tick resistance (BTA 20), and fecal larva count (BTA 21).

#### Prototheca spp.-positive enriched QTLs

A significant representation of *Milk* QTL class-related traits (15 out of 35) was also observed among those exclusively enriched in the P+ group (FDR < 0.05); as previously observed, the enriched traits were mainly related to milk fat and fatty acid composition, although specific traits were milk unsaturated fatty acid content and milk C17 index. Within the *Health* QTL class, clinical mastitis (BTA 17) was also specifically enriched in this group. All the traits exclusively enriched in P+ group are represented in Fig. 3b.

#### S. agalactiae-positive enriched QTLs

Once again, 24 out of the 54 traits exclusively enriched in the Sa+ group (FDR < 0.05; Fig. 3c) were associated with the *Milk* QTL class. Among these, milk potassium, omega-6 fatty acid, glycerophosphocholine, linoleic acid content, and β-lactoglobulin percentage were uniquely found in this group, regardless of chromosomal location. Bovine respiratory disease susceptibility (BTA 28), *M. paratuberculosis* susceptibility (BTA 3 and 23), and abomasum displacement were the health-related traits uniquely enriched in the Sa+ group.

#### sIMI groups enriched QTLs

**Fig. 4:**
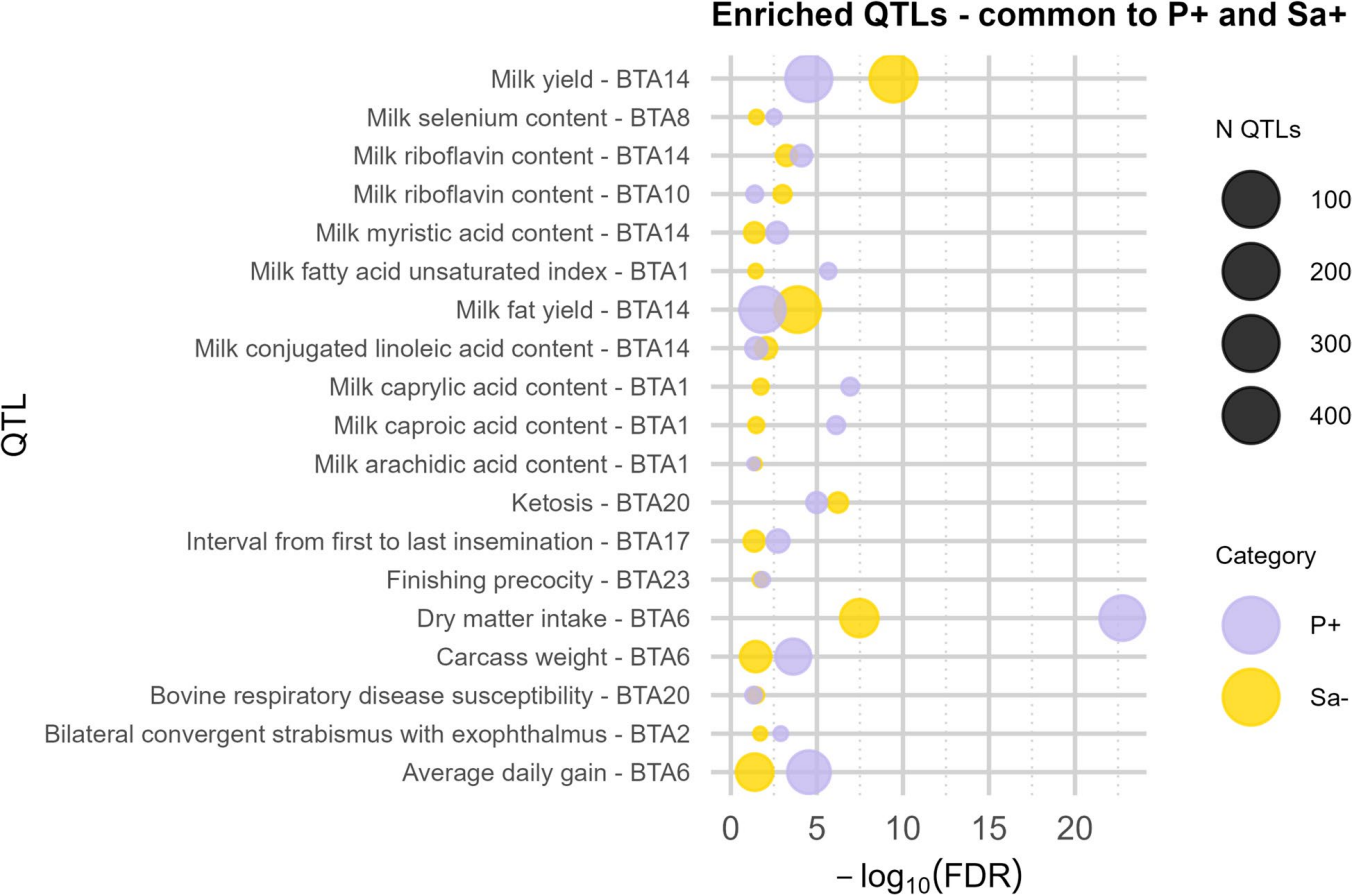
Enriched QTLs common to P+ and Sa+. Significantly enriched (FDR < 0.05) QTL traits co-localized with variants unique to *Prototheca* spp.-positive (P+), and *Streptococcus agalactiae*-positive (Sa+) group shared across both the positive groups. The area of the bubbles represents the number of observed QTLs for that trait. The *x*-axis shows the −log(*P*_adj_-value) scale, where *P*_adj_ refers to FDR. The *y*-axis reports the QTL trait and the corresponding *Bos taurus* chromosomes (BTA). Violet bubbles represent P+ group, yellow bubbles represent Sa+ group

A subset of 19 enriched traits (FDR < 0.05) was commonly shared by both the positive groups (P+ and Sa+) but not enriched in the negative one (Fig. 4). *Milk*-related traits, and in particular fatty acids content, were again predominant (i.e. Milk saturated fatty acid, caproic acid, arachidic acid content, and milk fatty acid unsaturated index). Bovine respiratory disease (BTA 20) and ketosis were the only enriched traits related to *Health* QTL class.

## Discussion

This study provides novel insights into the sequence variations present in the transcriptome of milk somatic cells in Holstein cattle under different intramammary infection conditions. We identified a distinct set of SNPs and INDELs whose presence at the transcriptomic level was associated with sIMI caused by *S. agalactiae* and *Prototheca* spp., compared to healthy controls. These findings suggest that pathogen-specific transcript-sequence variation may reflect differences in host immune response at the molecular level.

### Variant profile and predicted functional impact

Hundreds of thousands of variants were identified, with SNPs being significantly more abundant than INDELs. This result was expected, as INDELs are known to be less common in mammalian genomes [28, 29]. In fact, INDELs affecting coding regions are more likely to be deleterious and thus subject to stronger purifying selection, whereas SNPs tend to be more evenly distributed across both coding and non-coding regions [43].

Variants with predicted high-impact consequences were identified across all three investigated microbiological conditions. Importantly, these high-impact variants represented only a small fraction of the total detected variants, supporting the specificity of the prioritization strategy.

Notably, several of these variants, particularly INDELs, were located in immune-related genes that had already been identified as differentially expressed in previous analyses of the same dataset comparing the same experimental groups [33]. The identification of variants in immune-related differentially expressed genes was highly expected, as variant detection from RNA sequencing data is strongly influenced by gene expression levels and sequencing coverage. However, variants co-located with genes strongly expressed under specific microbiological conditions may have greater functional relevance, particularly when occurring in immune-related genes.

For instance, diverse variants uniquely detected in P+ and in Sa+ were predicted to have a high impact on TNFAIP3 interacting protein genes, such as TNFAIP3 interacting protein 1 (*TNIP1*) and TNFAIP3 interacting protein 3 (*TNIP3*). These proteins act like negative regulators of inflammatory and immune signaling, in particular through the repression of NF-κB pathway and modulation of Toll-like receptor signaling [44], thereby preserving tissues from immune-mediated damage [45]. Interestingly, the *TNIP1* transcripts carried a heterozygous frameshift insertion identified only in P+ group. Despite its potentially disruptive nature, gene expression in P+ remained unchanged compared with the negative group and was higher than in Sa+, as previously reported by Bisutti et al. [33] in the same experimental groups, suggesting no major effect on mRNA stability.

However, the predicted frameshift insertion may still affect the protein, potentially leading to partial loss of function. Given the role of *TNIP1* as a negative regulator of NF-κB signaling, even a moderate impairment of its activity could weaken inflammation. Such dysregulation may contribute to a sustained and poorly resolved inflammatory response, ultimately favoring the chronic, tissue-damaging nature of *Prototheca*-associated mastitis [46–48]. *TNIP1* has also been linked to mastitis resistance in dairy cattle [49], reinforcing its relevance as a candidate gene for further functional investigation. In *TNIP3* transcripts, a heterozygous splice donor SNP exclusively detected in the Sa+ group was identified. No alternative isoforms were detected in a previous isoform-level expression analysis [41], suggesting limited impact on alternative splicing, likely mitigated by the wild-type allele. However, its location at a splice donor site indicates potential functional relevance, as it may affect splicing efficiency, transcript processing and stability. *TNIP3* was found to be upregulated in the Sa+ group compared to the negative group [33], a pattern consistent with its role in inflammatory responses, as also reported by Cheng et al. [50] in cases of clinical mastitis. Given the stronger immune activation associated with *S. agalactiae* infections [51], regulatory variations within inflammation-related genes such as *TNIP3* may influence how the host modulates the inflammatory response. In this context, the splice donor SNP could contribute to fine-tuning *TNIP3* expression or activity during acute inflammatory stress, even in the absence of detectable structural transcript alterations.

Several immune-related loci included interleukin receptor genes, highlighting the centrality of cytokine signaling in shaping host responses to intramammary infections. Among them, *IL10RB*, encoding for interleukin 10 receptor subunit beta, is notable given the well-established role of IL-10 in dampening inflammation and promoting immune tolerance [52]. The co-located variant consists in a splice site donor SNP identified only in P+ group: while no major isoform changes were observed [41], due to the nature of splice donor variant, subtle effects on transcript stability or regulatory dynamics cannot be excluded. Interestingly, *IL10RB* expression was higher in P+ compared to Sa+ animals [33], which may reflect the chronic nature of *Prototheca*-associated mastitis [51] and support the modulatory effect of the SNP on IL10 signaling. Reinforcing its role, *IL10RB* was also identified as a candidate gene in a GWAS-based prioritization study for mastitis resistance traits in dairy cattle [10]. Other interleukin receptor genes, including *IL15RA* and *IL2RA*, were found to be co-located with splice donor variants uniquely detected in the negative condition. Both genes regulate immune cell activation and proliferation [53–55] and are typically upregulated during IMI [56, 57]. Consistently, higher expression levels in infected animals were previously reported by Bisutti et al. [33]. However, despite the lower expression, splice donor variants in *IL2RA* and *IL15RA* were detected exclusively in the Neg group, and no evidence of alternative isoforms were observed [41]. These variants may represent sequence alterations located at putative splicing regulatory sites, potentially influencing transcript processing or RNA stability. Nevertheless, the absence of detectable alternative isoforms suggests that any functional effect on splicing may be subtle or condition-dependent. Given the low basal expression of these genes under healthy conditions, the functional consequences of these variants may not be evidents and might emerge only upon immune challenge [58]. Overall, the identification of variants across multiple interleukin receptors highlights cytokine signaling as a potential regulatory hub in mastitis susceptibility. Rather than causing major isoform shifts, these variants may subtly modulate immune responses under different infectious contexts. Such plasticity within cytokine pathways may help explain the variability of mastitis outcomes and represents a promising avenue for further research. However, given the transcriptomic origin of these variants, the apparent group specificity should be interpreted cautiously. Nonetheless, their occurrence in genes involved in inflammatory regulation suggests a possible context-dependent biological relevance, although genomic and functional validation will be required to determine whether these variants contribute to differential immune responses.

### Variants effect on regulatory elements

Non-coding RNAs, including lncRNAs and miRNAs, represent key regulatory elements in the molecular networks controlling inflammatory responses, including those involved in mastitis [59–63]. The lncRNAs, for example, can modulate gene expression by interacting with adjacent target genes and acting through mechanisms such as transcriptional regulation, epigenetic modifications, and post-transcriptional control [61, 62, 64–66]. Moreover, when targeting immune-related genes, they may influence disease susceptibility and progression.

For instance, in this study we identified a SNP within the Neg group (G/A 20:70034262) overlapping the ENSBTAG00000050065, an annotated lncRNA previously found to be downregulated in mastitic animals compared to the healthy ones by Asselstine et al. [59]. The predicted target gene, located downstream the lncRNA, is the Iroquois homeobox 2 (*IRX2*), a gene which has been associated to tumor growth and metastasis [67]. However, no experimental or clinical evidence currently supports its involvement in mastitis, and its potential role warrants further investigation. Then, three distinct SNPs (rs379264194 and rs385867683 unique to P+, T/A 1:10845493 unique to Sa+) were identified in the region downstream the bta-miR-155. This miRNA has been demonstrated to play a dual role in response to *Mycobacterium tuberculosis* in murine model [68]: promoting macrophage survival, potentially favoring intracellular bacterial persistence, and enhancing T-cell mediated immune response. As previously mentioned, cytofluorimetric analyses revealed a higher proportion of macrophages in Sa+ samples, whereas P+ animals were lymphocyte-dominant [51]. This leukocyte distribution may reflect the dual functional role of bta-miR-155 across different immune cell types. Moreover, bta-miR-155 expression was also found upregulated in bovine macrophages upon *S. agalactiae* ST12 infection, correlating with stronger inflammation [69]. The presence of SNPs downstream of bta-miR-155 may influence regulatory elements involved in the control of miRNA transcription or processing, potentially modulating its expression. Such regulatory variability could contribute to pathogen-specific modulation of immune responses: in *S. agalactiae* infections, enhanced macrophage survival mediated by bta-miR-155 could support a more localized, acute inflammatory response, whereas in *Prototheca* spp. infections, increased T-cell activity may sustain a chronic immune activation state.

By acting as regulatory nodes within broader immune networks, these non-coding variants may influence the magnitude, timing, and specificity of immune responses, ultimately contributing to the variability of disease susceptibility and progression. Integrating genomic and functional analyses in future studies will be crucial to unravel how these elements orchestrate immune regulation and impact the complex genetic architecture underlying mastitis.

### QTL enrichment patterns

Mastitis resistance is a complex polygenic trait, influenced by multiple genes and regulatory elements, each contributing with a small effect [12]. Numerous studies have focused on unraveling its genetic architecture [10, 11, 13–16], but the findings are often inconsistent due to differences in population structure, environmental factors, genotyping platforms, and statistical approaches. As a result, association mapping approaches, such as GWAS, as well as sequencing-based variant discovery studies, typically identify dozens of loci, especially in non-coding regions, with modest effects and limited overlap across studies [10, 70]. To address this challenge, integrating functional genomic data, such as QTLs, with transcript-derived variants can provide additional biological context and help prioritize candidate regions [71].

The QTL annotation revealed a predominance of milk-related traits across all experimental groups, likely reflecting the overrepresentation of these QTLs in cattle databases due to the historical focus on milk production [29, 42]. Similar patterns were observed in studies investigating mastitis-associated candidate markers [10, 72], as well as in research on feed efficiency [29].

The enrichment analysis revealed that a substantial proportion of enriched QTLs remained associated with milk traits in all groups. This trend, also reported in previous studies on mastitis-related lncRNAs and gene expression [17, 59], may reflect both a residual bias and a true biological link. Indeed, genetic correlations between milk production and mastitis susceptibility are well documented [73, 74], supporting the hypothesis that a QTL influencing milk traits may also influence mastitis resistance, as suggested by other authors [74, 75]. This may be explained by pleiotropic effects, whereby a single genomic region controls the expression of multiple, seemingly unrelated phenotypes [17, 75].

An interesting finding was the enrichment of clinical mastitis related QTLs in BTA 17, which was uniquely observed analyzing *Prototheca* spp.-positive group. Although previous studies have mapped QTLs linked to clinical mastitis [11, 13, 15, 59, 70, 72, 76], none have specifically addressed QTL associations in mastitis cases caused by *Prototheca* spp. This result may reflect different dynamics:

the presence of variants associated with an increased risk of IMI and progression toward clinical mastitis, or, alternatively, the involvement of these variants in modulating host–pathogen interactions without necessarily determining clinical outcome, particularly considering that the animals included in the present study showed no clinical signs. These findings align with studies identifying specific chromosomal regions and copy number variations associated with clinical mastitis, often overlapping with immune-related genes [17, 77, 78]. Nevertheless, these variants were identified from RNA sequencing data, so it is more likely that they have a regulatory role in influencing the host immune response rather than being indicative of germline genetic predisposition.

Additional *Health*-related QTLs found to be enriched were ketosis, bovine respiratory disease susceptibility, *M. paratuberculosis* susceptibility, and bovine tuberculosis susceptibility. The first, ketosis, was common to both the IMI condition while absent in the healthy group, in accordance with the outcomes of other studies [10, 59, 72]. The presence of a positive, although low, genetic correlation between mastitis risk and metabolic diseases such as ketosis is well known [79, 80]. However, due to the transcriptome-origin of these variants, it is not possible to confirm their genomic origin or their potential contribution to genetic correlations between diseases. Instead, it is more likely that they are involved in regulatory processes affecting multiple disease-related pathways.

Similarly, the enrichment of bovine respiratory disease susceptibility QTLs found in this study is consistent with previous reports [10, 72]. Interestingly, this enrichment was detected in both infected and uninfected animals, suggesting that these genomic regions may have a broader role in immune regulation rather than being specific to IMI susceptibility. Overall, these findings support the hypothesis that shared immune-related loci may contribute to the modulation of host responses across multiple infectious conditions.

### Limitations

This study presents some limitations that should be considered when interpreting the results. First, variant identification was performed at the transcriptome level. Although sequences were pooled within microbiological conditions prior to variant calling, variant detection could have been influenced by gene expression levels, allele-specific expression, and sequencing depth, and the absence of a variant in a given condition does not necessarily indicate its genomic absence. The identified variants should thus be interpreted as condition-associated detected sequence variants (i.e. variants detected in specific microbiological conditions) rather than markers of genetic susceptibility. In addition, RNA was extracted from milk somatic cells, whose cellular composition may vary depending on the inflammatory status of the mammary gland, potentially affecting variant detection. Further genomic and functional validation, ideally in an independent population, will be necessary to confirm their genomic origin and clarify their potential biological and breeding relevance.

## Conclusions

In this study we highlight the value of a transcriptome-driven variant discovery approach to investigate the molecular basis of subclinical IMI. The identification of condition-associated SNPs and INDELs detected within the transcriptome, integrated with QTL information, provides novel insights into the immune response to sIMI and suggests potential genomic regions of interest.

In particular, variants detected in immune-related genes, as the *IL* receptor genes, may indicate a role for immune regulatory mechanisms in susceptibility to IMI and mastitis. At the same time, the presence of variants in non-coding regulatory regions such as lncRNAs and miRNAs suggests that transcriptional control and downstream gene modulation may be equally crucial in determining individual susceptibility. After a proper genomic and functional validation, these findings may provide a foundation for a more targeted breeding and disease management strategies, potentially leading to animals with greater resistance and improved control of IMI.

## Supplementary Information

Additional file 1: Table S1. Descriptive statistics of milk yield, milk composition, and udder health traits. Table S2. Variants uniquely identified in Negative group. Table S3. Variants uniquely identified in negative group and the predicted high impact consequence. Table S4. Variants uniquely identified in *Prototheca* spp.-positive group. Table S5. Variants uniquely identified in *Prototheca* spp. positive group and the predicted high impact consequence. Table S6. Variants uniquely identified in *Streptococcus agalactiae*-positive group. Table S7. Variants uniquely identified in *Streptococcus agalactiae*-positive group and the predicted high impact consequence. Table S8. Annotate QTLs within the 2 Kb interval from the candidate markers for Negative group. Table S9. Annotate QTLs within the 2 Kb interval from the candidate markers for *Prototheca* spp. Positive group. Table S10. Annotate QTLs within the 2 Kb interval from the candidate markers for *Streptococcus agalactiae*-positive. Table S11. Enriched QTLs within the 2 Kb interval from the candidate markers for Negative group. Table S12: Enriched QTLs within the 2 Kb interval from the candidate markers for *Prototheca* spp.-positive group. Table S13. Enriched QTLs within the 2 Kb interval from the candidate markers for *Streptococcus agalactiae*-positive group.

## Data Availability

The sequencing data of this study were deposited in the NCBI’s Sequence Read Archive (SRA) under the PRJNA911953 accession number.

## Abbreviations

DSCC: Differential somatic cell count
FDR: False discovery rate
IMI: Intramammary infection
INDELs: Insertions and deletions
QTL: Quantitative trait locus
SCC: Somatic cell count
SCS: Somatic cell score
Simi: Subclinical intramammary infection
SNP: Single nucleotide polymorphism
